# Adrenal Rests in the Uro-genital Tract of an Adult Population

**DOI:** 10.1007/s12022-021-09685-y

**Published:** 2021-06-07

**Authors:** Enrico Costantino Falco, Lorenzo Daniele, Jasna Metovic, Enrico Bollito, Giovanni De Rosa, Marco Volante, Mauro Papotti

**Affiliations:** 1 Pathology Unit, Department of Medical Sciences, Città Della Salute E Della Scienza Hospital, University of Turin, Turin, Italy; 2 Pathology Unit, Mauriziano Hospital, Turin, Italy; 3 Pathology Unit, Department of Oncology, Città Della Salute E Della Scienza Hospital, University of Turin, Turin, Italy; 4 Pathology Unit, San Luigi Hospital, Orbassano, Turin, Italy; 5 Pathology Unit, Department of Oncology at San Luigi Hospital, University of Turin, Orbassano, Turin, Italy

**Keywords:** Adrenal gland, Ectopia, Adrenal rests, Testis, Ovary, Neoplasia

## Abstract

Ectopic adrenal rests are a rare condition which can be found in various sites, generally in the retroperitoneum or pelvis along the path of gonadal descent. Their real prevalence is unknown. Males are more commonly affected, at least in the pediatric age. Adrenal rests are usually clinically silent and incidentally found in surgical samples, mostly in the pediatric population, and rarely in adults. With the aim of increasing knowledge and estimating the prevalence of ectopic adrenocortical tissue in the adult population, 44 adrenal rests in the urogenital tract of 40 adults are described. These represent approximately 0.07% of the total number of urogenital and gynecological surgeries performed in the 22 considered years. Adrenal rests were identified in the spermatic cord (10 males) and in paraovarian, parasalpingeal, or infundibulopelvic ligament locations (30 females). All but one was incidental findings. One case regarded an adrenocortical carcinoma arisen in adrenal rests. A literature review of adrenal ectopia in the urogenital tract of adults identified 57 reported cases from 53 patients, with similar clinicopathological features as those of our series, with the exception of a lower incidence of parasalpingeal locations. Despite their limited clinical implications, awareness of ectopic adrenal rests is essential also in adults for at least two reasons: (a) to correctly identify sources of adrenocortical hormone production in case of adrenal insufficiency or hormonal imbalance and (b) to avoid misinterpretations in the diagnostic workup of renal cell carcinoma, adrenocortical tumors, and rare gonadal neoplasms, including Sertoli/Leydig cell tumors.

## Introduction

The adrenal glands develop at 28–30 days after conception from two separate embryological tissues: the medulla derived from neural crest in proximity of dorsal aorta and the cortex from the intermediate mesoderm in the region between the genital ridge and the root of the mesentery [1]. During the 7th week, fusion between the two components takes place by migration and penetration of neural crest cells into the unencapsulated cortex [2]. At birth, the fetal cortex forms the largest part (70–85%) of the adrenal cortex, but it quickly undergoes vascular engorgement and atrophy. At the same time, the outer permanent cortex begins to differentiate into the definitive three layers [3–5].

Adrenal ectopia is defined as the presence of adrenal tissue in a location other than the adrenal glands, including the region around the adrenal gland, the celiac plexus, the kidney, and the route of gonadal descent, comprising also hernia sacs [6–10]. In exceptional cases, it can also be found in bizarre sites, such as placenta [11], lung [12], or intracranial cavity [13].

Ectopic adrenal nodules probably originate either from multiple primordia or from secondarily detached cortical fragments during the penetration of medullary cells into cortical anlage [14, 15]. Subsequently, these rests may remain close to the main gland or migrate, mostly in the pelvis or the groin region. The most frequent pelvic or groin location can be explained by the fact that the fetal adrenal cortex is often unencapsulated and develops in close contact with the gonads.

Ectopic nodules usually contain cortical components, only [5]. However, in the area of the celiac plexus, ectopic adrenal nodules have been reported to contain both cortex and medulla in about 50% of cases [16]. Ectopia in unusual anatomic sites is more difficult to explain, and other theories have been proposed, including a possible origin from pluripotent cells [14].

Macroscopically, ectopic adrenal tissue looks like a round, yellow, and well-defined node, usually smaller than 1 cm. Ectopic cortex shows normal morphology with the typical zonation, and responds to physiological ACTH stimulation. Therefore, although these rests are usually clinically silent, they may undergo hyperplasia in conditions associated with excessive adrenocorticotropic hormone (ACTH) production, such as in patients with Cushing’s disease or Nelson’s syndrome following bilateral adrenalectomy [17]. Ectopic adrenal nodules could also give rise to malignant tumors. Moreover, in congenital adrenal hyperplasia (CAH), an autosomal recessive disease that causes adrenal cortical dysfunction, the increased ACTH levels induce adrenocortical hyperplasia of orthotopic and heterotopic adrenal tissue [18].

The real incidence of adrenal rests is unknown, but they are reported to be much more frequent in infants than in adults. In intrauterine life, the adrenal glands are proportionally 20 times larger than those in adulthood, so ectopic tissue is also likely larger and more readily identified in infants and children. In fact, they are found in about 50% of newborns near the adrenal gland and in 7.5–15% of cases in the inguinal region, while their incidence drops to 1% in adults, as they are believed to degenerate or undergo atrophy within a few years [10, 14, 15].

The majority of ectopic adrenal tissues are described as incidental findings during surgical procedures in the urogenital tract of male children. Moreover, they are identified mostly by the pathologist in surgical material, whereas 14% of cases, only, are discovered by the surgeon, due to their small size and fat-like appearance [10]. In the adult population, adrenal ectopia is a rare occurrence.

Both in pediatric and in adult patients, the presence of ectopic adrenal tissue in the inguinal region is associated with undescended testis, probably because of a thorough dissection and careful examination of the spermatic cord. However, an alternative hypothesis suggest that inadequate or late migration of primordial germ cells could lead to closer interactions with adrenal cortical cells affecting the right location [19, 20].

Based on the above, the aim of the present study was to review a large retrospective series of adrenal ectopia in the urogenital tract in adults.

## Materials and Methods

**Fig. 1:**
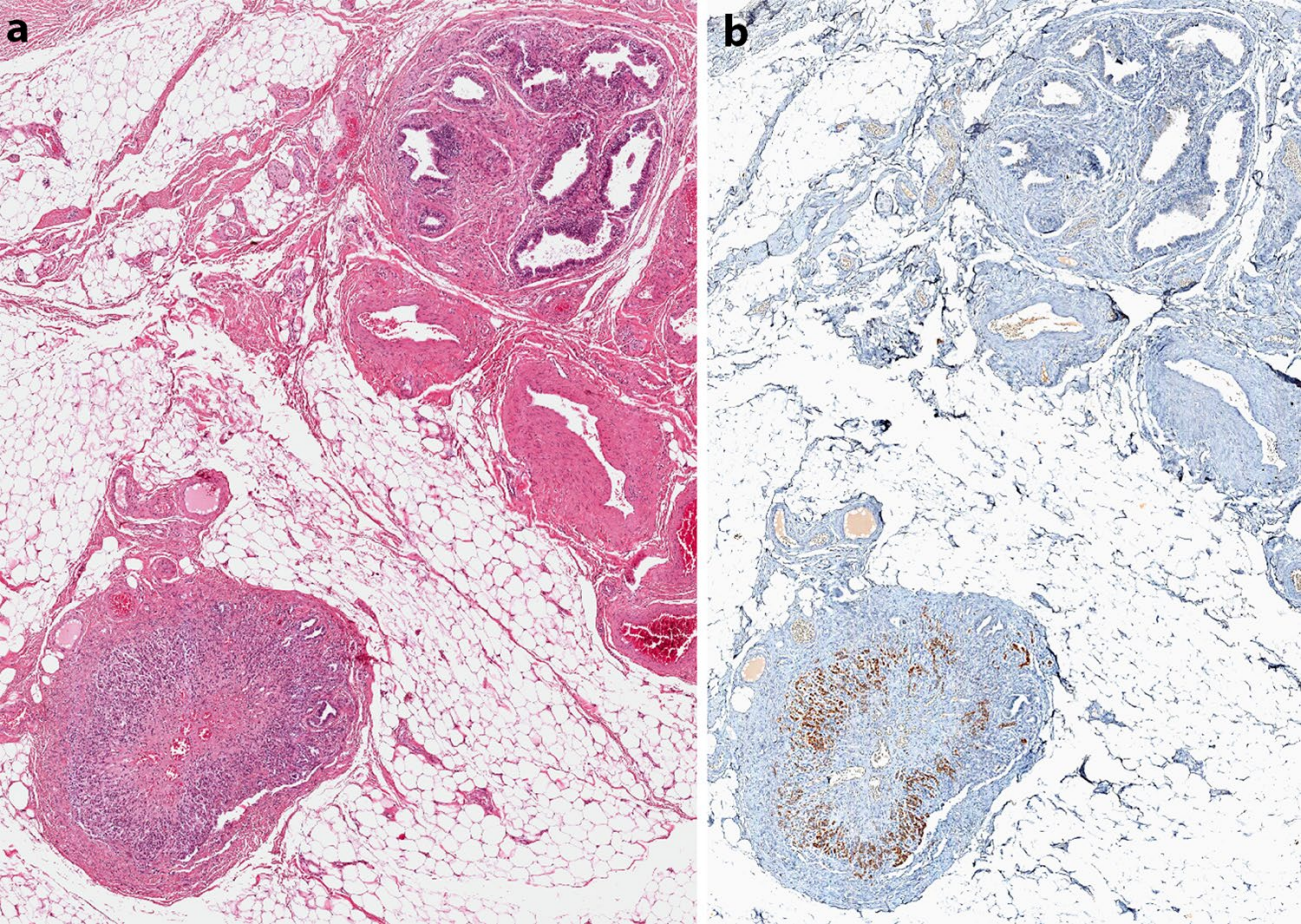
Case 15−Adrenal ectopia in the spermatic cord (original magnification × 50). Hematoxylin and eosin staining shows a well-defined nodule composed of cortical cells only and with a thin capsule in the adipose tissue close to the epididymis (**a**). Melan-A/MART-1 reactivity supported the diagnosis of an adrenal rest (**b**)

We retrospectively collected all cases of adrenal ectopia in the urogenital tract close to the gonads detected in surgically resected specimens of adult patients (age > 18 years) between 1999 and 2020 in three academic hospitals of Torino, Italy (Mauriziano, San Luigi and Città della Salute e della Scienza University Hospitals). In the considered timeframe, 53,983 urogenital and gynecological surgical specimens were submitted to histopathological examination. In particular, 39,286 and 14,697 surgical procedures were performed in women and in men, respectively. Histological reports were retrieved from the pathological databases of the three pathology divisions and were then integrated with data from the clinical and surgical databases. Data about sex, age, relevant medical history, reason for surgery, location, and morphological features of the ectopic rests were recorded. The study was approved by the Institutional Review Board of the San Luigi Hospital (Protocol AMPRECCO, No. 128/2010). After anonymization of pathology slides and blocks by a pathology staff member not involved in this study, all specimens were reviewed to confirm the diagnosis of ectopic adrenal tissue and to evaluate the specific histopathological features. In some cases, immunohistochemical stains for Melan-A/MART-1 (clone A103, Benchmark AutoStainer Ventana Medical Systems, AZ, USA) and for alpha-inhibin (clone R1, Ventana) were performed using a Ventana Roche immunohistochemistry platform (Fig. 1).

Moreover, the previous literature was reviewed to compare the morphological features of adrenal ectopia in the urogenital tract of the adult population. A search was set up in the PubMed database using the terms “ectopic adrenal,” “accessory adrenal,” “adrenal heterotopia,” “adrenal rests,” browsing titles, and abstracts in English language. All the articles reporting well-documented cases in adult patients (age > 18 years) and localized in the urogenital region near the gonads were included.

## Results

**Fig. 2:**
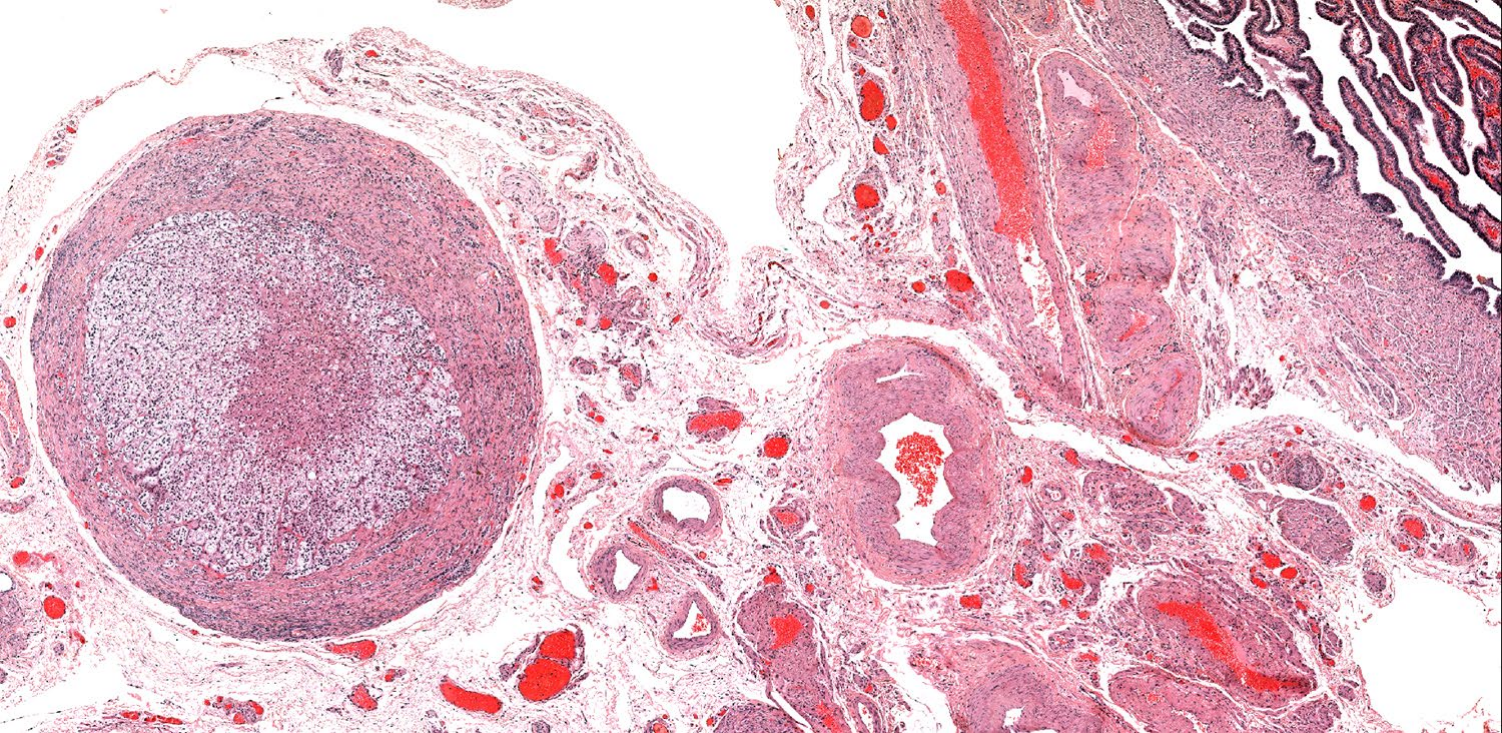
Case 40−Parasalpingeal adrenal rest stained with hematoxylin and eosin (original magnification × 50). A 2 mm adrenocortical nodule is located in the vascularized and loose connective tissue surrounding the salpinx

Forty cases of ectopic adrenal nodules in the urogenital region adjacent to the gonads were collected and reviewed. One case (No. 9) had already been described as part of a single case report of a testicular seminoma associated to adrenal rests [21], and case No. 14 was a consultation case reviewed by one of us (EB) (Fig. 2).

### General Information and Clinical Findings

Overall, adrenal rests were detected in 0.07% of urogenital tract surgical specimens in adults. The clinicopathological results are summarized in Table 1. Ectopic rests were found in 10 (25%) men and 30 women (75%), with a mean age of 54.6 years (range 26–86). Females had a mean age of 57.2 years, while males of 45.9 years. The nodules had a mean size of 3.0 mm (1–8 mm). In 37 patients, a single adrenal rest was identified, whereas multiple small nodules were found in three women, either in the adipose tissue close to the ovaries or in the infundibulopelvic ligament. The nodules were almost equally distributed on the right and left side (20 and 19, respectively; in one case, the information was missing), but with a difference between sexes. Indeed, a right-side preference was observed in men (6/9, 66.7%; missing information in one case), but not in women (14/30, 46.7%).

**Table 1:** Clinicopathological features of the 44 reported adrenal rests in the urogenital tract of 40 adults

No	Sex/Age	Cause of surgery	Adrenal rests			
			Laterality/Location	Macro/microscopic finding	Size (mm)	Histology
1	M/48	Cryptorchidism in single congenital kidney	L/Spermatic cord	Micro	4	Normal cortex
2	F/49	Uterine leiomyomas	NA/Parasalpingeal tissue	Micro	2	Normal cortex
3	F/75	Endometrial carcinoma	L/Paraovarian tissue	Micro	1	Normal cortex
4	F/65	Endometrial carcinoma	L/Parasalpingeal tissue	Micro	2	Normal cortex
5	F/46	Endometriotic cyst	NA/Parasalpingeal tissue	Micro	3	Normal cortex
6	F/68	Uterine prolapse	1R-2L/Paraovarian tissue	Micro	1 / 2 / 2	3 nodules, normal cortex
7	F/47	Prophylactic adnexectomy	R/Paraovarian tissue	Micro	3	Normal cortex
8	M/56	Inguinal hernia and cryptorchidism	R/Spermatic cord	Micro	3	Normal cortex
9	M/43	Seminoma	R/Spermatic cord	Macro	3	Normal cortex
10	F/32	Ovarian cystadenoma	R/Ovarian hilus	Micro	2	Normal cortex
11	M/37	Mixed malignant germ cells tumor	R/Spermatic cord	Micro	1	Normal cortex
12	M/28	Mixed malignant germ cells tumor	R/Spermatic cord	Macro	3	Normal cortex
13	F/52	Ovarian cystadenofibroma	L/Paraovarian tissue	Micro	2	Normal cortex
14	M/NA	Inguinal mass	NA/ Spermatic cord	Macro	NA	Carcinoma
15	M/29	Seminoma	L/Spermatic cord	Micro	2	Normal cortex
16	M/26	Mixed malignant germ cells tumor	R/Spermatic cord	Micro	4	Normal cortex
17	F/60	Pelvic leiomyosarcoma	NA/Paraovarian tissue	Micro	5	Normal cortex
18	F/50	Endometrial stromal sarcoma	L/Parasalpingeal tissue	Micro	8	Normal cortex
19	F/55	Ovarian carcinoma	Peritoneum L/paracolic gutter	Micro	3	Normal cortex
20	F/67	Salpingeal adenocarcinoma	R/Infundibulopelvic ligament	Micro	4	Normal cortex
21	F/61	Endometrial carcinoma	R/Infundibulopelvic ligament	Micro	3 / 5	2 nodules, normal cortex
22	F/71	Bladder urothelial carcinoma	L/Paraovarian tissue	Micro	4	Normal cortex
23	F/85	Ovarian borderline cystadenoma	NA/Parasalpingeal tissue	Micro	5	Normal cortex
24	F/55	Vaginal carcinoma	R/Parasalpingeal tissue	Micro	3	Normal cortex
25	F/52	Ovarian carcinoma	R/Infundibulopelvic ligament	Micro	2 / 2	2 nodules, normal cortex
26	F/54	Endometrial carcinoma	L/Parasalpingeal tissue	Micro	3	Normal cortex
27	F/54	Endometriotic cyst	R/Parasalpingeal tissue	Micro	2	Normal cortex
28	F/56	Ovarian cystadenoma	L/Ovarian hilus	Micro	2	Normal cortex
29	F/75	Endometrial carcinoma	L/Infundibulopelvic ligament	Micro	1	Normal cortex
30	F/51	Adult granulosa cell tumor	L/Parasalpingeal tissue	Micro	2	Normal cortex
31	F/86	Bladder urothelial carcinoma	R/Parasalpingeal tissue	Micro	3	Normal cortex
32	F/60	Ovarian cystadenoma	R/Parasalpingeal tissue	Micro	1	Normal cortex
33	F/41	Cervical adenocarcinoma	R/Parasalpingeal tissue	Micro	3	Normal cortex
34	M/80	Spermatocytic tumor	L/Parafunicular fat	Micro	3	Normal cortex
35	F/57	Endometrial carcinoma	L/Parasalpingeal tissue	Micro	4	Normal cortex
36	M/66	Prostate adenocarcinoma	R/Parafunicular fat	Micro	5	Normal cortex
37	F/46	Salpingitis	L/Parasalpingeal tissue	Macro	6	Normal cortex
38	F/39	Salpingo-ovarian abscess	L/Paraovarian tissue	Micro	4	Normal cortex
39	F/60	Metastatic gastric carcinoma	R/Parasalpingeal tissue	Micro	2	Normal cortex
40	F/48	Uterine leiomyomas	L/Parasalpingeal tissue	Micro	2	Normal cortex

*F* female, *M* male, *R* right, *L* left, *NA* not available

All but one case was clinically silent, and none of them was recognized by surgeons during excision: in three cases, ectopic nodules were incidentally discovered at the time of macroscopic dissection in the pathology laboratory, while most of them were recognized at the time of microscopic analysis, only. None of the patients had primary adrenal diseases or symptoms related to hormonal excess, and no hormonal deficits resulted from the excision of the accessory tissue. Among males, the ectopic nodules were found in the spermatic cord after surgery performed for hernia repair, prostate carcinoma, cryptorchidism, or testicular tumors, while in females, they were discovered in paraovarian or paratubal location after surgery for gynecological neoplasms or bladder carcinoma. Case No. 14 was a carcinoma strictly related to adrenal rests identified in the periphery of the tumor and close to the testis. The patient underwent surgery for inguinal pain, and multiple nodules were found along the spermatic cord. A hepatic node was also excised, whereas no abnormalities were detected in the adrenal glands. Benign rests in males were associated with testicular malignancy (six cases), prostate adenocarcinoma (one case), and with undescended testis (the remaining two cases). Interestingly, in one of such cases (#1), cryptorchidism was associated with a congenital solitary kidney, ipsilateral to the cryptorchid testis. Among females, fourteen cases were associated with gynecological malignancies (two leiomyosarcomas, six endometrial, four ovarian/salpingeal, one cervical, and one vaginal carcinomas), three cases with extra-gynecological malignancies (two bladder and one gastric carcinomas), and one borderline ovarian tumor. The other twelve cases were associated with benign gynecological conditions including four ovarian cystadenomas, two leiomyomas, two endometriotic cysts, two salpingitis, and one uterine prolapse, the remaining case being a prophylactic bilateral adnexectomy in a BRCA mutated breast carcinoma patient.

### Pathological Findings

Macroscopically, adrenal rests presented as circumscribed round to oval small nodules, with a tan-yellow color, and different in consistency from the surrounding adipose tissue. The microscopic analysis revealed well-defined nodules composed of adrenal cortex, only, surrounded by a thin fibrous capsule. They had all a comparable morphology, with cells arranged in two or three layers. Zona fasciculata was predominant in all cases, followed by zona reticularis. Glomerulosa cells were present in small clusters beneath the capsule in larger nodules and absent in smaller ones. The cells had a cord-like arrangement with a minimal interposed stroma, and were large and polygonal, with abundant vacuolated granular and eosinophilic cytoplasm and small nuclei. Conversely, in both testicular and hepatic lesions of case No. 14, a solid proliferation of moderately atypical polygonal cells with a high mitotic count and foci of necrosis was identified. A diagnosis of adrenocortical carcinoma in ectopic adrenal rests with liver metastasis was rendered in this case. In addition to the routine hematoxylin and eosin, immunostaining for Melan-A (in case 11, 15, 16, 29, and 30) and for alpha-inhibin (in case 13) were also performed, confirming the adrenocortical nature of the tissue. Notably, all cases in our series were correctly identified in the original diagnosis as ectopic adrenal cortical tissue, irrespective of the location and pathology associated.

### Review of the Literature

The English literature review identified 37 articles reporting 57 ectopic adrenal nodules in the urogenital tract (excluding the kidney) of 53 adult patients (27 men and 26 women). Demographic and clinic-pathological data are summarized in Table 2. Almost all studies are single-case reports (33/37 papers), with the largest series being those by Falls and Gutowski (11 and 5 cases, respectively) [6, 16]. The mean age was 41.8 years (range 19–78 years). Females had a mean age of 38.2 years, while males of 45.5 years. The nodules had a mean size of 15 mm (range 0.4–145 mm). In 3 patients (2 females and 1 male), bilateral nodules were found, while two ectopic rests were located in the same hernia sac in a man. In the other 49 patients, a single adrenal rest was identified. Overall, the majority of nodules were identified on the right side (36/56, 64.3%; the information was not available in one case). Among men, ectopic tissue was found in the spermatic cord, in hernia sacs, and in paratesticular location. In women, the most frequent locations were the broad ligament and the paraovarian region, followed by the wall of ovarian cysts and a single case in the appendiceal tissue adherent to mesovarium. Only the nodule found in the wall of an ovarian serous cystadenoma had both adrenal cortical and medullary components, while in all the other cases, in both males and females, adrenal cortical tissue, only, was identified. The majority of cases were asymptomatic and incidentally found after surgical exploration of the pelvic region for different reasons. Of the 54 cases with this information available, 42 were recognized at the time of surgical excision or during the macroscopic gross evaluation of the specimen and 12 were identified at the microscopic analysis. Nine cases were associated to an adrenocortical tumor, namely, six clinically silent adenomas and three carcinomas with Cushing’s syndrome.

**Table 2:** Clinicopathological features of 57 adrenal rests reported in literature in the urogenital tract of 53 adults

No	Author [reference]	Sex/Age	Cause of surgery	Adrenal rests			
				Laterality/Location	Macroscopic/microscopic finding	Size (mm)	Histology
1	Gualtieri [22]	M/31	Scrotal mass	L/Spermatic cord	Macro	40	Adenoma
2	Janovski [23]	F/42	Cervical carcinoma	R/Mesosalpinx	Macro	15	Choristoma
3	Schechter [14]	M/19	Inguinal hernia	L/Hernia sac	Macro	2	Normal cortex
4	Morimoto [24]	M/57	Scrotal mass in Cushing’s syndrome	L/Paratesticular	Macro	50	Carcinoma
5	Gutowski [6]	M/58	Inguinal hernia	R/Hernia sac	Micro	4	Normal cortex
6	Gutowski [6]	M/66	Inguinal hernia	R/Hernia sac	Macro	10	Normal cortex
7	Gutowski [6]	M/44	Inguinal hernia	R/Hernia sac	Macro	2 / 5	2 nodules, normal cortex
8	Gutowski [6]	M/45	Inguinal hernia	L/Hernia sac	Macro	3	Normal cortex
9	Gutowski [6]	M/34	Inguinal hernia	R/Hernia sac	Micro	2	Normal cortex
10	Anderson [15]	F/62	Acute appendicitis	R/Appendiceal mesentery	Macro	10	Normal cortex
11	Anderson [15]	M/53	Inguinal mass	R/Spermatic cord	Macro	10	Normal cortex
12	Lodeville [25]	M/21	Nodule of the spermatic cord	R/Spermatic cord	Macro	1.5	Normal cortex
13	Czaplicki [26]	M/34	Cryptorchidism and sterility	R/Paratesticular	Macro	5	Adenoma
14	Czaplicki [26]	M/34	Cryptorchidism and sterility	L/Paratesticular	Micro	NA	Normal cortex
15	Van Ingen [27]	F/41	Testosterone-producing tumor	L/Mesovarium	Macro	40	Adenoma
16	Sasano [28]	F/43	Uterine leiomyoma	R/Broad ligament	Macro	50	Adenoma
17	Ventura [29]	M/42	Scrotal trauma	R/Spermatic cord	Macro	5	Normal cortex
18	Mari [21]	M/43	Seminoma	R/Spermatic cord	Macro	3	Normal cortex
19	Usta [30]	F/21	Ovarian cystadenoma	L/Ovary	Macro	2	Normal cortex and medulla
20	Iyengar [8]	M/54	Inguinal hernia	R/Hernia sac	Micro	0.4	Normal cortex
21	Ors [31]	F/44	Paraovarian mass	R/Paraovarian	Macro	20	Adenoma
22	Jain [32]	M/65	Testicular masses in Cushing’s syndrome	R-L/Paratesticular	Macro	31 / 85	2 nodules, carcinoma
23	El Demellawy [7]	M/78	Inguinal hernia	L/Hernia sac	Macro	7	Normal cortex
24	Yasar [33]	F/50	Granulosa cell tumor	L/Ovary	NA	NA	Normal cortex
25	Rabie [34]	M/26	Undescended testis	L/Paratesticular	Micro	NA	Normal cortex
26	Zhong [35]	F/56	Ovarian cystadenoma	R/Paraovaric	Micro	2	Normal cortex
27	Müllhaupt [36]	M/44	Varicocele	NA/Spermatic cord	Macro	9	Normal cortex
28	Floyd [37]	M/45	Seminoma in undescended testis	R/Spermatic cord	Macro	5	Normal cortex
29	Takeuchi [38]	M/52	Lipoma of spermatic cord	R/Spermatic cord	Micro	NA	Normal cortex
30	Kasajima [39]	F/29	Pelvic mass in oligomenorrhea	L/Broad ligament	Macro	65	Adenoma
31	Niveditha [40]	M/64	Inguinal hernia	R/Hernia sac	Macro	4	Normal cortex
32	Raman [41]	M/28	Undescended testis	R/Paratesticular	Micro	2	Normal cortex
33	Sangeeta [42]	M/21	Undescended testis	R/Paratesticular	Macro	NA	Normal cortex
34	Khandakar [43]	F/26	Ovarian cystadenoma	R/Paratubal	Micro	6	Normal cortex
35	Chentli [44]	F/34	Pelvic mass in Cushing’s syndrome	R/Ovary	Macro	145	Carcinoma
36	Senescende [10]	M/44	Inguinal hernia	R/Hernia sac	Macro	10	Normal cortex
37	Billone [45]	F/22	R/hydrosalpinx and torsion	L/Infundibulopelvic ligament	Macro	3	Normal cortex
38	Chew [46]	F/22	Ovarian cystadenoma	L/Ovary	Micro	NA	Normal cortex
39	Kassaby [47]	M/56	Inguinal hernia	L/Hernia sac	Micro	1.5	Normal cortex
40	Sahin [48]	F/65	Ovarian cystadenoma	L/Ovary	NA	NA	Normal cortex
41	Tingi [49]	F/48	Dysmenorrhea	L/Fallopian tube	NA	NA	Normal cortex
42	Alimoradi [50]	M/37	Bilateral inguinal hernia	R/Hernial sac	Micro	NA	Normal cortex
43	Falls [16] 13 cases in 11 consecutive pts	F/35.7	Endometrial carcinoma, leiomyomas, polycystic ovaries, ruptured tubal	8R, 1L, 2 Bilateral/Broad ligament	Macro	2 (1–4)	Normal cortex

*F* female, *M* male, *R* right, *L* left, *NA* not available, *pts* patients

## Discussion

In this study, we reported a series of 44 adrenal rests found in the peri-gonadal location of the urogenital tract in an adult population of 40 patients, with a female predominance. A PubMed review of the English literature disclosed 57 published cases, for a total of 101 ectopic adrenal nodules in the path of gonadal descent, supporting the notion that adrenal ectopia is a rare, but not exceptional, occurrence in adults.

After birth, this accessory tissue undergoes involution, and in the presence of a normal hormonal function of the main glands, the ectopic nodes are believed to undergo complete atrophy and eventually disappear [14]. During the atrophic process, small adrenal rests are relatively common in the pediatric age, especially in male children undergoing urogenital surgery or hernia repair, often in the presence of congenital adrenal hyperplasia.

In adults, small adrenal rests may occasionally remain close to urogenital tract organs, and in exceptional conditions of hormonal deficits or abnormal stimulation, they could increase in size and become the source of adrenocortical hormone production. Adrenal rests are usually identified in specimens of gynecological surgery or in the context of inguinal hernia repair or orchidopexy procedures [14, 26, 34]. In the present case series, undescended testis in male patients was the unique non-neoplastic associated pathology, with 2 out of 10 cases. Interestingly, the association of adrenal ectopia with cryptorchidism and ipsilateral congenital solitary kidney in case #1 highlights the embryological relationship between fetal adrenal and genitourinary structures. Renal agenesis is reported to be associated with absence or ectopia of adrenal gland in about 10% of cases [51], and cryptorchidism could be associated with minor or major urinary tract abnormalities such as single kidney, unilateral renal hypoplasia, ureterocele, or ureteric stenosis, usually ipsilateral to the undescended testis [52]. However, in our case, the urological anomaly was contralateral to the cryptorchid testis (left undescended testis and right kidney agenesis). No other cases of ectopic adrenal tissue associated with undescended testis and concomitant renal agenesis have been described in adults, so far. One case in a man was a primary adrenocortical carcinoma from ectopic adrenal tissue in the spermatic cord, associated with liver metastases. A literature search revealed only three previous cases of carcinoma arising in ectopic adrenal rests of the urogenital tract in the adult population, all associated with Cushing’s syndrome [24, 32, 44]. The remaining seven male patients were affected by testicular (six cases) or prostatic (one case) malignancies. To the best of our knowledge, only three cases associated with testicular tumors in adults are on record in the English literature [21, 25, 37].

In female patients, the majority of surgical specimens derived from hysterectomies or ovariectomies, with a remarkable finding of adrenal rests in ovarian and salpingeal ligaments (9 and 16 cases, respectively), the remaining cases being located in the infundibulopelvic ligament or paracolic tissue.

In our adult series of adrenal ectopia, the sex distribution and overall anatomical sites, including the absence of a predominant right location, are opposed to those of the literature. A possible explanation could be that in the pediatric population, surgical procedures more frequently are performed in males and involve the groin region, leading to a higher detection of clinically silent adrenal rests along the spermatic cord or rete testis [10, 19, 53, 54]. Indeed, in adults, gonadal resection is more likely to occur in women, thus explaining the higher number of adrenal rests in female patients of the current series. However, if we compare the number of ectopic nodules with the number of surgical procedures performed in the same time interval, we get the same incidence rate (0.07%) in both sexes. A very old study on a consecutive series of hysterectomies showed that an extensive sampling of the broad ligaments allowed to identify adrenal rests (having a median size of 2 mm) in up to 25% of cases [16]. Therefore, the evaluation of the exact incidence and sex distribution of adrenal rests in the general population and in adults in particular, seems to suffer from numerous biases, precluding a precise estimate.

Regarding right-side predominance of adrenal ectopia, this is well documented in the male pediatric population, but not in adults, due to the lack of large series [10, 19]. Considering the currently reported cases, the right-side preferential location seems to occur also in adults, especially in male patients, being negligible in females. In any case, a selection bias linked to the type of pathology specimens analyzed cannot be excluded: in fact, inguinal hernias are the main reason for groin region exploration in children and they typically develop far more commonly on the right side than in the left [55]. Moreover, also cryptorchidism and testicular cancer are reported to have a right-side predominance and, therefore, adrenal rests may well be incidentally detected in the same location [56].

Adrenal medulla was not found in any of the cases of the current series and only in one case among those described in the literature, with an overall prevalence of 1% (1/101). This finding contrasts with the data of some early literature that reported the presence of medulla in up to 50% of cases of adrenal rests in the celiac-plexus area [16]. This discordance may be explained by a different time of adrenal tissue displacement, with the nodules in the celiac-plexus area, localized close to the adrenal glands, arising in a late phase of embryonic development (after the fusion between cortex and medulla), while those found far away being displaced at early (pre-fusion) stages.

Regarding hormonal function of adrenal rests, although such ectopic tissue is generally devoid of clinical relevance, it is to be noticed that it is hormonally active and able to respond to external stimuli. Thus, as also reported in animal models, after adrenocortical failure or injury, compensatory hormonal function of ectopic rests has been reported [16, 57]. ACTH-mediated hyperplasia of accessory nodules could also account for refractory Cushing’s syndrome, replacing adrenal hormonal production [58]. On the other hand, adrenal insufficiency can follow ectopic tissue excision, if this was the only functional adrenal tissue of the patient [10].

All but one (the ectopic adrenocortical carcinoma) currently reported case was clinically silent and incidentally found. Nevertheless, tumors arising from adrenal rests are on record, often benign and non-functioning. In a few cases, ectopic tissue may be directly responsible for Conn’s syndrome [59] or ACTH-independent Cushing ‘s syndrome, due to adenomas [27, 28, 39, 60, 61] and exceptionally to carcinomas [32, 62, 63]. Indeed, adenoma is not easily differentiated from normal ectopic tissue, except for an aberrant hormonal production or a large size. Carcinomas are conversely more easily recognizable, based on the Weiss score parameters, as observed in the single malignant case of our series. Other neoplasms of gonadal origin including steroid producing tumors [44, 64–66] have a gross and microscopic resemblance to adrenocortical tumors, determining differential diagnosis problems from overgrown adrenal rests, although the location of gonadal neoplasms is intraparenchymal, while adrenal rests generally occur in the periphery of the gonad [67]. In addition, in the rete testis of CAH patients, adrenal rests can expand in up to 40% of cases leading to the so called testicular adrenal rest tumor (TART), that can cause infertility because of compressive/obstructive events [68, 69]. Such TART nodules are functionally and histologically similar to adrenocortical tissue, and may result from ectopic adrenal cell proliferation or from a totipotent embryonic cell type in the testis [5].

In conclusion, the actual prevalence of ectopic adrenocortical rests in the adult population is probably much higher than reported, but the low clinical relevance and the small size of ectopic nodules cause that most of them are not recognized. Awareness of the occurrence of ectopic adrenal tissue is important because of hyperplasia and even neoplastic transformation of such rests in patients with symptoms caused by hypersecretion of adrenocortical hormones, which can well be ectopically produced. In addition, ectopic adrenal tissue must be differentiated from urogenital tumors, in particular its mimickers, such as renal cell carcinoma and Sertoli Leydig cell tumor, that also partially share the immunophenotypic markers of the adrenal cortex (Melan-A, SF-1, inhibin). Indeed, misinterpretation of ectopic adrenal tissue is unlikely, and morphology and appropriate immunohistochemistry are adequate for addressing a correct diagnosis in most cases.

## Data Availability

The datasets generated and/or analyzed during the current study are not publicly available due to privacy reasons but are available from the corresponding author on reasonable request.
